# Metacognitions About Smoking: Psychometric Properties of the Italian Version of the Metacognitions About Smoking Questionnaire

**DOI:** 10.1002/cpp.70208

**Published:** 2025-12-22

**Authors:** Sara Palmieri, Ana Nikčević, Gabriele Caselli, Tatiana Marci, Claudia Marino, Marcantonio M. Spada, Giovanni Mansueto

**Affiliations:** ^1^ Department of Psychology Sigmund Freud University Milan Italy; ^2^ Cognitive Psychotherapy School and Research Center, Studi Cognitivi Milan Italy; ^3^ Department of Psychology, School of Law, Social and Behavioural Sciences Kingston University Kingston UK; ^4^ Department of Developmental and Social Psychology University of Padova Padua Italy; ^5^ School of Applied Sciences London South Bank University London UK; ^6^ Department of Health Sciences University of Florence Florence Italy

**Keywords:** cigarette, emotional distress, metacognitions, Metacognitions about Smoking Questionnaire, nicotine, smoking

## Abstract

This study aimed to evaluate the factor structure, internal consistency and concurrent validity of the Italian version of the Metacognitions about Smoking Questionnaire (MSQ), utilizing the framework of the Self‐Regulation Executive Function model. A total of 532 smokers completed the Italian‐translated version of the MSQ, the Fagerstrom Test for Nicotine Dependence, the Severity Dependence Scale, the Depression Anxiety and Stress Scale‐21. To test the factorial structure of the MSQ, a series of confirmatory factor analyses (CFAs) were run; correlational analyses and structural equation modelling (SEMs) approaches were undertaken to evaluate the concurrent validity. The 12‐item MSQ with four factors was confirmed: positive metacognitions about cognitive regulation (PM‐CR), positive metacognitions about emotional regulation (PM‐ER), negative metacognitions about uncontrollability (NM‐U) and negative metacognitions about cognitive interference (NM‐CI). The MSQ showed an overall satisfactory fit index (TLI = 0.949, CFI = 0.963, RMSEA = 0.082 [0.064–0.101]). Internal consistency was satisfactory. MSQ factors are associated with higher nicotine/cigarette dependence and emotional distress, supporting the concurrent validity of the tool. Specific metacognitions about smoking were associated with different clinical outcomes related to smoking. A higher engagement on PM‐CR and on NM‐U was positively associated with nicotine/cigarette dependence. A higher engagement on PM‐ER and NM‐CI was associated with greater emotional distress. The 12‐item Italian version of the MSQ could be a promising tool to assess specific metacognitions about smoking in both research and clinical contexts. Metacognitions about smoking may be a suitable therapeutic target to reduce the levels of nicotine/cigarette dependence and emotional distress among smokers.

## Summary

- The Italian version of the MSQ is a suitable clinical tool that may help clinicians assess specific metacognitions about smoking.
- Higher endorsement of metacognitions about smoking may be associated with more severe nicotine/cigarette dependence.
- Higher endorsement of metacognitions about smoking may be associated with more severe emotional distress.
- Assessing metacognitions about smoking may allow clinicians to gain a clearer understanding of treatment trajectories among smokers.
- Metacognitions about smoking may be a suitable therapeutic target to maximize clinical outcomes among smokers.

## 1 Introduction

### 1.1 The Global Burden of Smoking

Smoking is one of the major global health problems (WHO 2023). It is the second‐leading risk factor for early death and disability and among the 10 largest contributors to global disability‐adjusted life years (GBD 2015^2016^; Reitsma et al. 2021). It is expected that by 2030, around 7.4 million deaths will be attributable to tobacco smoking (WHO 2008). Despite compelling evidence on the health hazards of smoking (GBD 2016; WHO 2023), for most smokers, quitting is a difficult process (Moffatt et al. 2004; Pereira et al. 2020; WHO 2023). It is estimated that the rates of smoking cessation are usually around 40% at the end of treatment of nicotine dependence and 25%–30% after 1 year of follow‐up (Lancaster et al. 2000). Therefore, exploration of maintenance factors underlying smoking is important as it may allow for the identification of modifiable factors that could be targeted in clinical interventions (Reitsma et al. 2021; WHO 2023).

### 1.2 Metacognitions as Potential Maintenance Factors of Smoking

Among cognitive and behavioural models, the Self‐Regulation Executive function (S‐REF) model (Wells and Matthews 1996) may be able to offer insights into potential maintenance factors of smoking (Spada et al. 2007; Nikčević and Spada 2008). According to the S‐REF model (Wells and Matthews 1996), psychological disorders and addictive behaviours are linked to maladaptive metacognitions (Spada et al. 2007; Wells and Matthews 1996), which refer to the information that individuals hold about their own cognition and about coping strategies that impact on it (Wells and Matthews 1996). Metacognitions can be divided into two broad sets: (1) positive metacognitions about control strategies that impact on inner events (e.g., ‘Rumination will help me get things sorted out in my mind’ and ‘If I worry I will be prepared’) (Wells 2000) and (2) negative metacognitions concerning the significance, controllability and danger of inner events (e.g., ‘It is bad to have certain thoughts’ and ‘I cannot stop ruminating’) (Wells 2000). Metacognitions have been found to be associated with a wide array of psychological disorders such as affective disorders, obsessive–compulsive disorder, eating disorders, psychosis, emotion and behaviour dysregulation (Mansueto et al. 2022, 2025; Mansueto, Jarach, Caselli, et al. 2024; Mansueto, Palmieri, Caselli, and Spada 2024; Mansueto, Palmieri, Sassaroli, et al. 2024; Olivari et al. 2025; Palmieri et al. 2021, 2024; Palmieri, Mansueto, et al. 2023; Palmieri, Sassaroli, et al. 2023; Sun et al. 2017; Wells and Matthews 1996) and addictive behaviours such as alcohol use, substance use, gambling, problematic internet and smartphone use (Casale et al. 2021; Mansueto et al. 2016; Mansueto, Palmieri, Caselli, and Spada 2024; Mansueto, Palmieri, Sassaroli, et al. 2024; Marino et al. 2025; Hamonniere and Varescon 2018; Spada et al. 2008, 2015).

Within the area of smoking, two main lines of research have explored the association between metacognitions and smoking. The first line of research, by using the Metacognitions Questionnaire‐30 (Wells and Cartwright‐Hatton 2004), has explored the association between generic metacognitions and smoking (Nikčević and Spada 2008; Spada et al. 2007). Spada et al. (2007) have found evidence that positive beliefs about worry, negative beliefs about worry concerning uncontrollability and danger and beliefs about cognitive confidence were correlated with more severe smoking dependence. Spada et al. (2008) found that high‐ and low‐dependency smokers endorsed stronger beliefs about the need to control thoughts when compared to non‐smokers, and that high‐dependency smokers endorsed stronger positive beliefs about worry when compared with non‐smokers. They also found an independent contribution, over negative emotions, of beliefs about the need to control thoughts towards category membership as a nicotine‐dependent smoker (Nikčević and Spada 2008).

In view of the findings from the above studies, a second line of research has been carried out to explore the existence of specific metacognitions about smoking (Nikčević and Spada 2010; Nikčević et al. 2015). In 2010, Nikčević and Spada undertook a qualitative study identifying specific positive and negative metacognitions about smoking. Positive metacognitions about smoking have been conceptualized as a specific form of outcome expectancy that may be motivating individuals to engage in smoking as a means of cognitive‐emotional regulation (e.g., ‘Smoking helps me to think things through’ and ‘Smoking helps me to feel less pressure’) (Nikčević and Spada 2010). Negative metacognitions about smoking have been conceptualized as beliefs concerning the uncontrollability of smoking and smoking‐related thoughts (e.g., ‘I cannot stop thinking about cigarettes’), and the perceived negative impact of smoking on self‐appraisal and cognitive functioning (e.g., ‘Smoking is a sign of my low will power’) (Nikčević and Spada 2010). Negative metacognitions about smoking are thought to play a crucial role in the perpetuation of smoking by becoming activated during and following a smoking episode and triggering negative emotional states that are likely to lead to smoking more (Nikčević and Spada 2010).

### 1.3 The Metacognitions About Smoking Questionnaire (MSQ)

On the basis of findings obtained from Nikčević and Spada (2010), Nikčević et al. (2015) developed a measure to assess specific metacognitions about smoking, that is, the MSQ. The MSQ is a 20‐item self‐reported questionnaire, which is rated on a 4‐point Likert scale (Nikčević et al. 2015). Using exploratory and confirmatory factor analyses (CFAs), Nikčević et al. (2015) demonstrated that the MSQ comprises four factors, positive metacognitions about cognitive regulation (e.g., ‘Smoking helps me think more clearly’) (PM‐CR), positive metacognitions about emotional regulation (e.g., ‘When I get stressed smoking calms me down’) (PM‐ER), negative metacognitions about uncontrollability of smoking and smoking‐related thoughts (e.g., ‘It is hard to control my desire for cigarettes’) (NM‐U) and negative metacognitions about cognitive interference (e.g., ‘My preoccupation with cigarettes takes over my life’) (NM‐CI). The MSQ has been shown to possess good concurrent and predictive validity, internal consistency and temporal stability (Nikčević et al. 2015). The MSQ has been validated across different cultures. Alma et al. (2018) validated the MSQ in a sample of adult Turkish smokers demonstrating an adequate fit of the four‐factor structure, along with good internal consistency and concurrent validity showing that metacognitions about smoking predicted nicotine dependence over and above demographic variables, length of cigarette use, negative affect and smoking outcome expectancies. Najafi et al. (2018) validated the MSQ in a sample of adult Iranian male smokers showing that four‐factor structure of the MSQ Persian version had appropriate fit and good reliability. Najafi et al. (2018) also reported that the MSQ Persian version had good discriminant validity showing that the MSQ subscale significantly differentiates Iranian smokers with higher scores on positive/negative smoking outcome expectancies from those with lower scores. In addition, Najafi et al. (2018) found that the MSQ Persian version had good predictive validity showing that, over and above smoking outcome expectancies, PM‐ER were positively associated with daily cigarette use, and both NM‐U and NM‐CI were positively associated with nicotine dependence. Sumbe et al. (2024) validated the MSQ among US adolescent and young adult e‐cigarette users, proposed a three‐factor structure of the MSQ US version (i.e., PM‐CR, PM‐ER and negative metacognitions) reporting a good internal consistency and a significant association between the three factors of the MSQ US version and past 30‐day e‐cigarette use. Overall, the MSQ could be a reliable measure of metacognitions about smoking.

### 1.4 Aims of the Study

Accumulative evidences showed that a higher endorsement of metacognitions about smoking is related to more severe smoking outcomes such as a higher number of daily cigarette use, higher nicotine dependence, higher urge to smoke, more severe withdrawal symptoms and higher emotional distress (Alma et al. 2018; Izadpanah et al. 2021; Khosravani et al. 2024; Najafi et al. 2018; Nikčević et al. 2015; Poormahdy et al. 2022; Turliuc et al. 2024). In addition, emerging research indicates a potential distinction in how metacognitions relate to addictive behaviours over different timeframes. Metacognitions related to addictive behaviours may be differentially associated with immediate, short‐term outcomes, such as craving, versus more enduring, long‐term consequences, such as dependence (Izadpanah et al. 2021; Poormahdy et al. 2022; Khosravani et al. 2021; Khosravani et al. 2024). Findings from smoking‐related research suggested that positive metacognitions are linked to heightened craving, whereas negative metacognitions are more strongly associated with dependence (e.g., Izadpanah et al. 2021; Poormahdy et al. 2022; Khosravani et al. 2024).

Based on this evidence, assessing and addressing metacognitions about smoking may be important during the process of smoking cessation in order to maximize clinical outcomes (Khosravani et al. 2024; Najafi et al. 2018; Nikčević et al. 2015). The MSQ (Nikčević et al. 2015) appears to be a promising tool to enable clinicians to assess specific positive and negative metacognitions about smoking (Alma et al. 2018; Najafi et al. 2018; Nikčević et al. 2015; Sumbe et al. 2024). Only a few studies have explored the psychometric properties of the MSQ (Alma et al. 2018; Najafi et al. 2018; Nikčević et al. 2015, Sumbe et al. 2024). So far, no research has evaluated the factor structure, validity and reliability of the MSQ in an Italian population. Considering the high prevalence of smoking in Italy (around 24%; ISS 2024) and a lack of instruments for the assessment of metacognitions about smoking, we set out to examine the psychometric properties of the Italian version of the MSQ (Nikčević et al. 2015). Based on the extant literature (Alma et al. 2018; Khosravani et al. 2024; Najafi et al. 2018; Nikčević et al. 2015), we hypothesized that (a) the Italian version of the MSQ would have a four‐factor structure and satisfactory internal consistency; (b) metacognitions about smoking would be associated with higher nicotine dependence; (c) metacognitions about smoking would be associated with higher emotional distress.

## 2 Method

### 2.1 Participants

This was a cross‐sectional study, employing a sample of 532 individuals residing in Italy and self‐identifying as ‘cigarette smokers’. Inclusion criteria were as follows: (1) 18 years of age or above; (2) understand spoken and written Italian; (3) self‐identified ‘smoker’ (i.e., subjects smoking regular cigarettes); and (4) consented to participate in the study. The mean age of the participants was 36.03 years (SD = 11.69); 356 (66.92%) were female, and 176 (33.08%) were male. Out of 532 participants, 412 (77.44%) were employed, 53 (9.96%) were students, 20 (3.76%) were housewives, 21 (3.95%) were retired and the remaining 26 (4.89%) were unemployed. Participants' mean scores on daily cigarette use, on the Fagerstrom Test for Nicotine Dependence (FTND; Heatherton et al. 1991), on the Severity Dependence Scale (SDS) (Gossop et al. 1995, 1997) and age of inception of cigarette use were, respectively, 11.8 cigarettes (SD = 7.44), 3.4 (SD = 2.22), 1.58 (SD = 0.62) and 17.03 years (SD = 3.93).

Ethics approval for the study was obtained from Sigmund Freud University Milan branch (n° ID7WD2GHC6VC1F91138). All procedures contributing to this work complied with the ethical standards of the relevant national and institutional committees on human experimentation and with the Helsinki Declaration of 1975, as revised in 2008.

### 2.2 Procedure

The original MSQ (Nikčević et al. 2015) was translated into Italian utilizing the forward and backward‐translation method (Sousa and Rojjanasrirat 2011). First, the MSQ was translated into Italian by two bilingual independent translators. Second, the Italian version was back translated into English by another two bilingual independent translators. A comparison was made between the forward and backward translations of the measure to evaluate for any discrepancy of sentence structure and ensure the translation's accuracy. Discrepancies were examined with the collaboration of the authors of the MSQ (Nikčević et al. 2015). Then, a pilot testing of the final version of the measure was tested in 10 smokers volunteer subjects. Participants were asked about the clarity and understandability of the measure. There were no apparent issues regarding the clarity of the measure, and no changes were required. Thereafter, the final version of the Italian MSQ was administered to the participants in our study.

### 2.3 Measures

MSQ is a 20‐item self‐report measure assessing metacognitions about smoking (Nikčević et al. 2015). It consists of four subscales: PM‐CR, PM‐ER, NM‐U and NM‐CI. The items are rated on a 4‐point Likert scale (from 1 *do not agree* to 4 *agree very much*). Higher scores indicate more dysfunctional metacognitions about smoking. The MSQ has shown good psychometric properties (Alma et al. 2018; Najafi et al. 2018; Nikčević et al. 2015; Sumbe et al. 2024).

FTND (Heatherton et al. 1991) is a six‐item self‐report measure assessing nicotine dependence. A total FTND score can be obtained by the addition of scores for all items. Higher scores denote higher levels of nicotine dependence. The Italian version of the FTND has been shown to possess good psychometric properties (Fekketich et al. 2008).

SDS (Gossop et al. 1995, 1997) is a five‐item scale evaluating the severity of cigarette dependence (Grassi et al. 2014). The SDS has 5 items: ‘Do you think your use of cigarette is out of control?’ (Item 1), ‘Does the prospect of missing cigarette making you very anxious or worried?’ (Item 2), ‘Do you worry about your use of cigarettes?’ (Item 3), ‘Do you wish you could stop smoking?’ (Item 4) and ‘How difficult did you find it to stop, or go without smoking?’ (Item 5). All items are scored on a 4‐point scale. Items 1–4 are scored as 0 = *never*, 1 = *sometimes*, 2 = *often* and 3 = *always*, whereas Item 5 is scored as 0 = *not difficult*, 1 = *quite difficult*, 2 = *very difficult* and 3 = *impossible* (Gossop et al. 1995, 1997). A total SDS score can be obtained by the average of items score. Higher scores indicate a greater degree of cigarette dependence. The Italian version of the SDS has been shown to possess good psychometric properties (Grassi et al. 2014).

Depression, Anxiety and Stress Scale‐21 (DASS‐21) (Lovibond and Lovibond 1995) is a 21‐item self‐report measure to assess general emotional distress experienced during the past week. The DASS‐21 is composed of three subscales: depression, anxiety and stress. The items are rated on a 4‐point Likert scale (from 0 *not all* to 3 *most of the time*). Higher scores indicate higher levels of depression, anxiety and stress. The Italian version of DASS‐21 has been shown to possess good psychometric properties (Bottesi et al. 2015; Iannattone et al. 2024; Lovibond and Lovibond 1995) and appropriate clinimetric properties (Mansueto et al. 2023).

### 2.4 Statistical Analyses

Analyses were carried out using R (R Development Core Team 2021). First, we computed and evaluated the main descriptive statistics and examined the item response distribution using the *psych* package (Revelle 2025). Construct validity was evaluated through a multistep approach. First, we tested the factorial structure of the instrument. As the MSQ is a theory‐based measure of metacognitions about smoking (Nikčević et al. 2015), grounded in the well‐established metacognitive model (Wells and Matthews 1996; Wells 2000) and supported by previous research (e.g., Alma et al. 2018; Najafi et al. 2018), we adopted a confirmatory approach with a semi‐exploratory purpose (Marci et al. 2020). Specifically, a series of CFAs were conducted while maintaining an exploratory approach using the *cfa* function included in the *lavaan* package (Rosseel 2012). Item loadings and modification indices were carefully inspected, and items presenting multiple issues (e.g., cross‐loadings or multicollinearity with other items) were systematically evaluated for their content and, when necessary, removed through a sequential procedure. All models were estimated using the *cfa* function included in the *lavaan* package (Rosseel 2012) with the Robust Diagonally Weighted Least Squares Mean and Variance (WLSMV) estimator, recommended for ordinal data (Brown 2006; Rhemtulla et al. 2012). Models were evaluated using multiple fit indices, including the chi‐square to degrees‐of‐freedom ratio (χ^2^/df), the root mean square error of approximation (RMSEA), the comparative fit index (CFI), the Tucker–Lewis index (TLI) and the standardized root mean square residual (SRMR). Based on Schermelleh‐Engel et al.'s (2003) guidelines, a CFI and TLI greater than 0.95, an RMSEA below 0.08 and an SRMR below 0.10, were considered indicators of a good model fit.

In the second step, we examined the average variance extracted (AVE). In line with the guidelines of Hair et al. (2022), AVE values equal to or greater than 0.50 were considered acceptable indicators of convergent validity. To assess discriminant validity among the latent dimensions, the Heterotrait–Monotrait (HTMT) ratio of correlations was used (Henseler et al. 2015). HTMT values below 0.85 or a more lenient threshold of 0.90 are generally considered indicative of discriminant validity. HTMT values below 0.85 or a more lenient threshold of 0.90 are generally considered indicative of discriminant validity.

Concurrent validity, as an indicator of criterion validity, was assessed by examining the relationships between the scale scores and a set of theoretically related external constructs (Alma et al. 2018; Khosravani et al. 2024; Najafi et al. 2018; Nikčević et al. 2015). First, at the univariate level, Pearson's correlations were calculated between MSQ's dimensions and external variables. Then, at the multivariate level, four separate structural equation models (SEMs) were estimated using the *sem* function included in the *lavaan* package (Rosseel 2012). Specifically, the MSQ latent dimensions were considered as predictors (exogenous variables), whereas the FTND (M1), SDS (M2), DASS subscales (M3) observed score were considered as outcomes (endogenous variables). Additionally, a further model was tested, including the MSQ latent dimensions as exogenous variables and all endogenous variables simultaneously (M4). In all models, gender was included as a covariate. Models were evaluated considering several fit indices and interpreted following the guidelines reported earlier. Association between MSQ dimensions and criteria measures was evaluated in terms of their significance and the strength of the associations. A value of *r* = 0.10 to be a small effect, *r* = 0.30 a medium effect and *r* = 0.50 a large effect (Cohen 1988).

In terms of reliability, internal consistency was evaluated using ordinal Cronbach's alpha and McDonald's omega calculated based on the CFA model by using the function reliability included in the *semTools* package (Jorgensen et al. 2025).

## 3 Results

### 3.1 Descriptive Analysis

The item response distributions and the main descriptive statistics for each item are reported in Table 1. The mean scores for the items range from 1.43 to 2.96. The presence of valuable skewness in some items (i.e., > |1.00|) further supports the use of WLS as the estimator method for CFA.

**TABLE 1:** Descriptive analyses.

MSQ items	*n*	Mean	SD	Median	Min	Max	Range	Skew	Kurtosis	SE
MSQ‐1	532	1.93	0.92	2.00	1.00	4.00	3.00	0.52	−0.84	0.04
MSQ‐5	532	2.02	0.92	2.00	1.00	4.00	3.00	0.45	−0.79	0.04
MSQ‐9	532	2.02	0.92	2.00	1.00	4.00	3.00	0.50	−0.71	0.04
MSQ‐13	532	1.92	0.92	2.00	1.00	4.00	3.00	0.63	−0.64	0.04
MSQ‐17	532	2.12	0.99	2.00	1.00	4.00	3.00	0.45	−0.89	0.04
MSQ‐2	532	2.96	0.93	3.00	1.00	4.00	3.00	−0.44	−0.83	0.04
MSQ‐6	532	2.93	0.94	3.00	1.00	4.00	3.00	−0.36	−0.95	0.04
MSQ‐10	532	2.67	0.99	3.00	1.00	4.00	3.00	−0.11	−1.06	0.04
MSQ‐14	532	2.52	0.96	2.00	1.00	4.00	3.00	0.02	−0.94	0.04
MSQ‐18	532	2.43	0.94	2.00	1.00	4.00	3.00	0.14	−0.86	0.04
MSQ‐3	532	2.04	1.09	2.00	1.00	4.00	3.00	0.61	−0.99	0.05
MSQ‐7	532	2.52	1.05	3.00	1.00	4.00	3.00	−0.04	−1.20	0.05
MSQ‐11	532	1.80	1.04	1.00	1.00	4.00	3.00	1.01	−0.34	0.05
MSQ‐15	532	1.88	1.02	2.00	1.00	4.00	3.00	0.84	−0.53	0.04
MSQ‐19	532	1.99	1.04	2.00	1.00	4.00	3.00	0.69	−0.75	0.04
MSQ‐4	532	1.55	0.90	1.00	1.00	4.00	3.00	1.54	1.21	0.04
MSQ‐8	532	1.90	1.04	2.00	1.00	4.00	3.00	0.76	−0.75	0.05
MSQ‐12	532	1.43	0.84	1.00	1.00	4.00	3.00	1.91	2.57	0.04
MSQ‐16	532	1.45	0.83	1.00	1.00	4.00	3.00	1.78	2.08	0.04
MSQ‐20	532	1.55	0.86	1.00	1.00	4.00	3.00	1.48	1.20	0.04

*Note:* *N* = 532.

Abbreviation: MSQ = Metacognitions about Smoking Questionnaire.

### 3.2 Factor Structure

**FIGURE 1:**
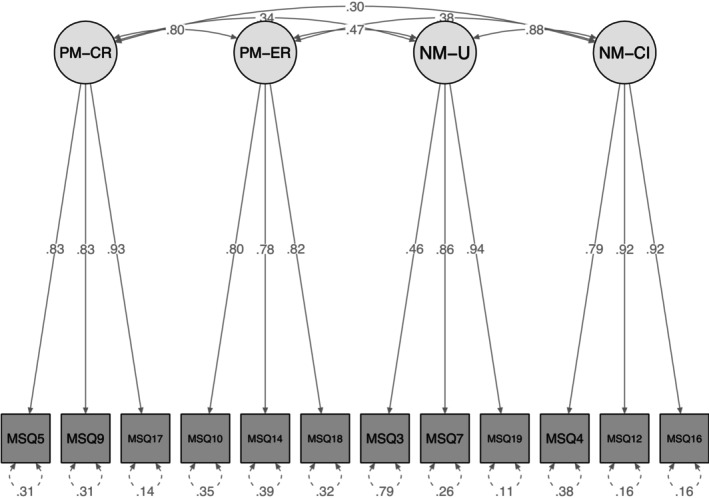
Confirmatory factor model of the 12‐items MSQ. *Note:* All structural coefficients are standardized. All factor loadings are significant at the 0.05 level. NM‐CI = negative metacognitions about cognitive interference, NM‐U = negative metacognitions about uncontrollability, PM‐CR = positive metacognitions about cognitive regulation, PM‐ER = positive metacognitions about emotional regulation.

The initial four‐factor model including all 20 items showed an unsatisfactory fit (Table 2). All factor loadings were found to be good, indicating strong relationships between the observed indicators (items) and their respective latent variables. However, an inspection of the modification indices revealed several items exhibiting multiproblematic aspects (i.e., multicollinearity and/or cross‐loadings). First, by adopting a conservative approach, we included the covariance of three pairs of items. Despite the improvements in the model (Table 2), there were still items showing more than one problematic issue. We then eliminated, following a step‐by‐step procedure, seven items that exhibited cross‐loadings on more than one factor and/or multicollinearity. CFA based on the remaining 13 items showed a good fit (Table 2). However, to yield a more parsimonious version of the instrument, an additional item was removed. The final model showed good fit indexes, and all standardized factor loadings were high and significant at the 1% level (*p* < 0.001) (Table 2 and Figure 1) (Appendices A and B).

**TABLE 2:** Model fit for the confirmatory factorial analyses.

	Notes	TLI_Robust_	CFI_Robust_	RMSEA [90% CI] _Robust_
Model 1–4 factors	—	0.775	0.806	0.155 [0.145–0.166]
Model 2–4 factors	Include covariance between MSQ_3 ~~ MSQ_11	0.865	0.884	0.121 [0.11–0.131]
Model 3–4 factors	Include covariance between MSQ_2 ~~ MSQ 6	0.884	0.901	0.112 [0.102–0.122]
Model 4–4 factors	Include covariance between MSQ_4 ~~ MSQ_20	0.894	0.910	0.107 [0.096–0.117]
Model 5–4 factors	Removed MSQ_1	0.903	0.919	0.105 [0.094–0.116]
Model 6–4 factors	Removed MSQ_2 and covariance between MSQ_2 ~~ MSQ_6	0.901	0.918	0.107 [0.096–0.119]
Model 7–4 factors	Removed MSQ_6	0.902	0.920	0.098 [0.110–0.122]
Model 8–4 factors	Removed MSQ_8	0.938	0.923	0.099 [0.086–0.113]
Model 9–4 factors	Removed MSQ_20 (and consequently covariance between MSQ_4 ~~ MSQ_20)	0.933	0.947	0.094 [0.080–0.109]
Model 10–4 factors	Removed MSQ_11 (and consequently covariance between MSQ_3 ~~ MSQ_11)	0.938	0.952	0.091[0.075–0.107]
Model 11–4 factors	Removed MSQ_13	0.949	0.962	0.083 [0.066–0.101]
Model 12–4 factors	Removed MSQ_15 (per arrivare allo stesso numero di item per dimensione)	0.949	0.963	0.082 [0.064–0.101]

*Note:* *N* = 532.

Abbreviations: CFI = Comparative Fit Index, RMSEA = Root Mean Square of Approximation, SRMR = Standardized Root Mean Square Residual, TLI = Tucker–Lewis Index.

The analysis of AVE values indicated good levels of convergent validity across all dimensions. Specifically, AVE scores were 0.748 for PM‐CR, 0.643 for PM‐ER, 0.616 for NM‐U and 0.765 for NM‐CI. All values were well above the commonly accepted threshold of 0.50, suggesting that each dimension explains a substantial portion of the variance in its observed indicators.

As reported in Table S1, the HTMT values for all dimension pairs were below the commonly accepted threshold of 0.85, with the only exception being the association between NM‐U and NM‐CI, which showed a value of 0.894. While this is slightly above the conservative cutoff, it still falls within the more flexible threshold of 0.90 considered acceptable within the social sciences research (Henseler et al. 2015).

### 3.3 Concurrent Validity and Internal Consistency

**FIGURE 2:**
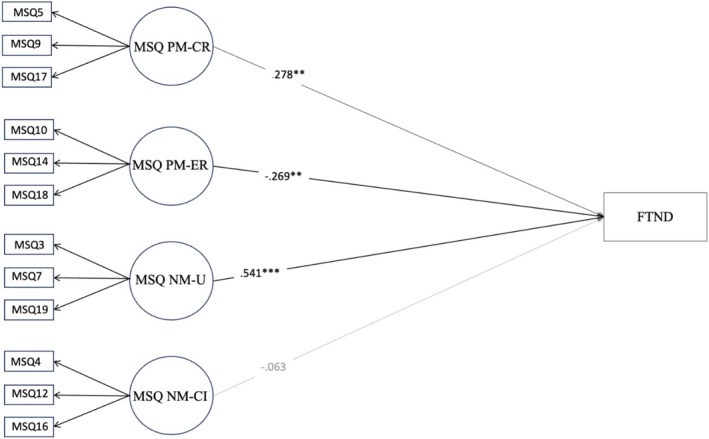
Structural equation model of the association between the MSQ four factors model and FTND nicotine dependence. *Note: N* = 532. All structural coefficients are standardized. Model fit: TLI = 0.969, CFI = 0.977, RMSEA = 0.045 [0.034–0.056]. FTND = Fagerstrom Test for Nicotine Dependence, MSQ NM‐CI = Metacognitions about Smoking Questionnaire—Negative metacognitions about cognitive interference, MSQ NM‐U = Metacognitions about Smoking Questionnaire—Negative metacognitions about uncontrollability, MSQ PM‐CR = Metacognitions about Smoking Questionnaire—Positive metacognitions about cognitive regulation, MSQ PM‐ER = Metacognitions about Smoking Questionnaire—Positive metacognitions about emotional regulation. Outcome was controlled for gender.

**FIGURE 3:**
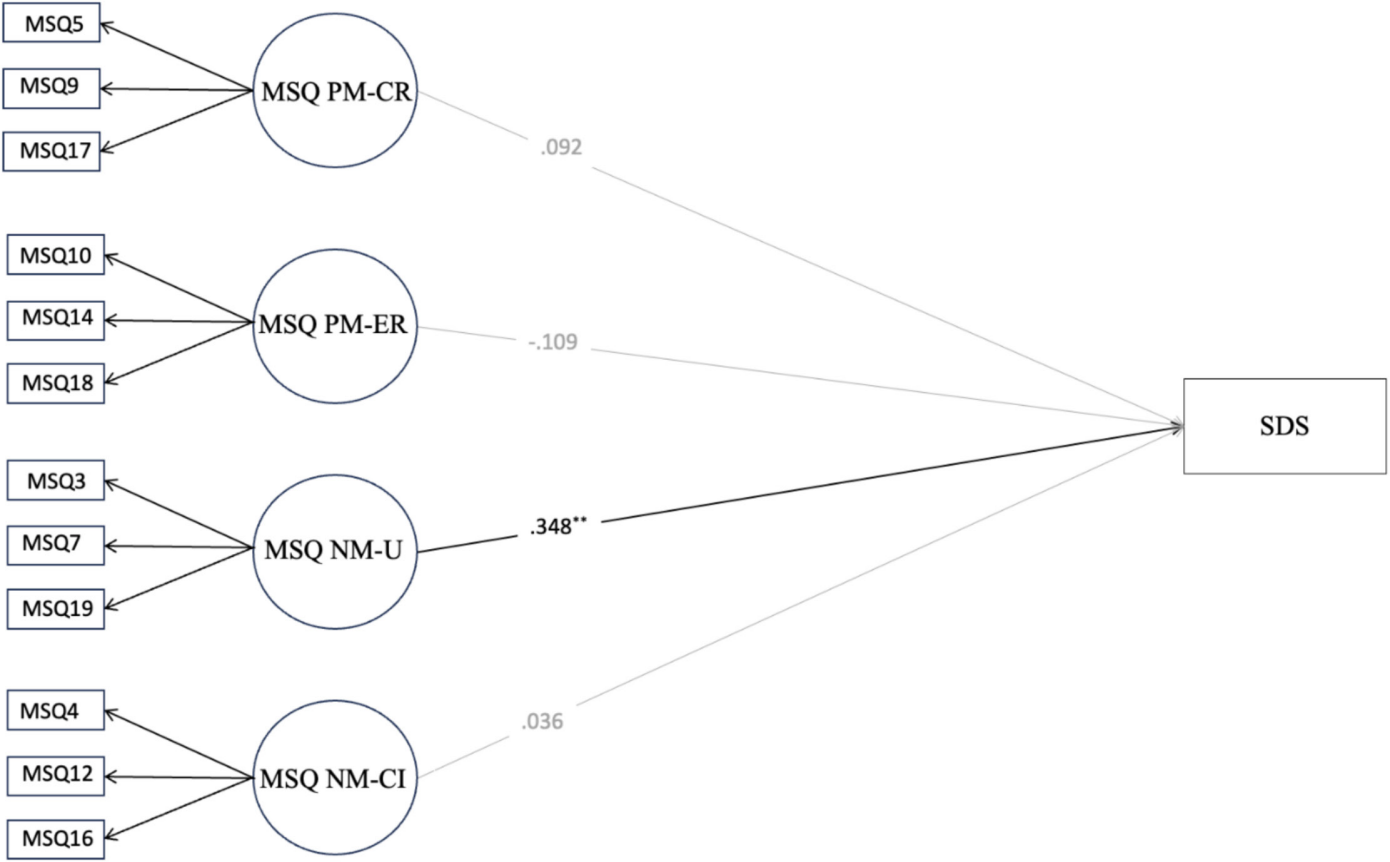
Structural equation model of the association between the MSQ four factors model and severity of SDS cigarette dependence. *Note: N* = 532. All structural coefficients are standardized. Model fit: TLI = 0.962, CFI = 0.971, RMSEA = 0.050 [0.039–0.061]. MSQ NM‐CI = Metacognitions about Smoking Questionnaire—Negative metacognitions about cognitive interference, MSQ NM‐U = Metacognitions about Smoking Questionnaire—Negative metacognitions about uncontrollability, MSQ PM‐CR = Metacognitions about Smoking Questionnaire—Positive metacognitions about cognitive regulation, MSQ PM‐ER = Metacognitions about Smoking Questionnaire—Positive metacognitions about emotional regulation, SDS = Severity of dependence scale. Outcome was controlled for gender.

**FIGURE 4:**
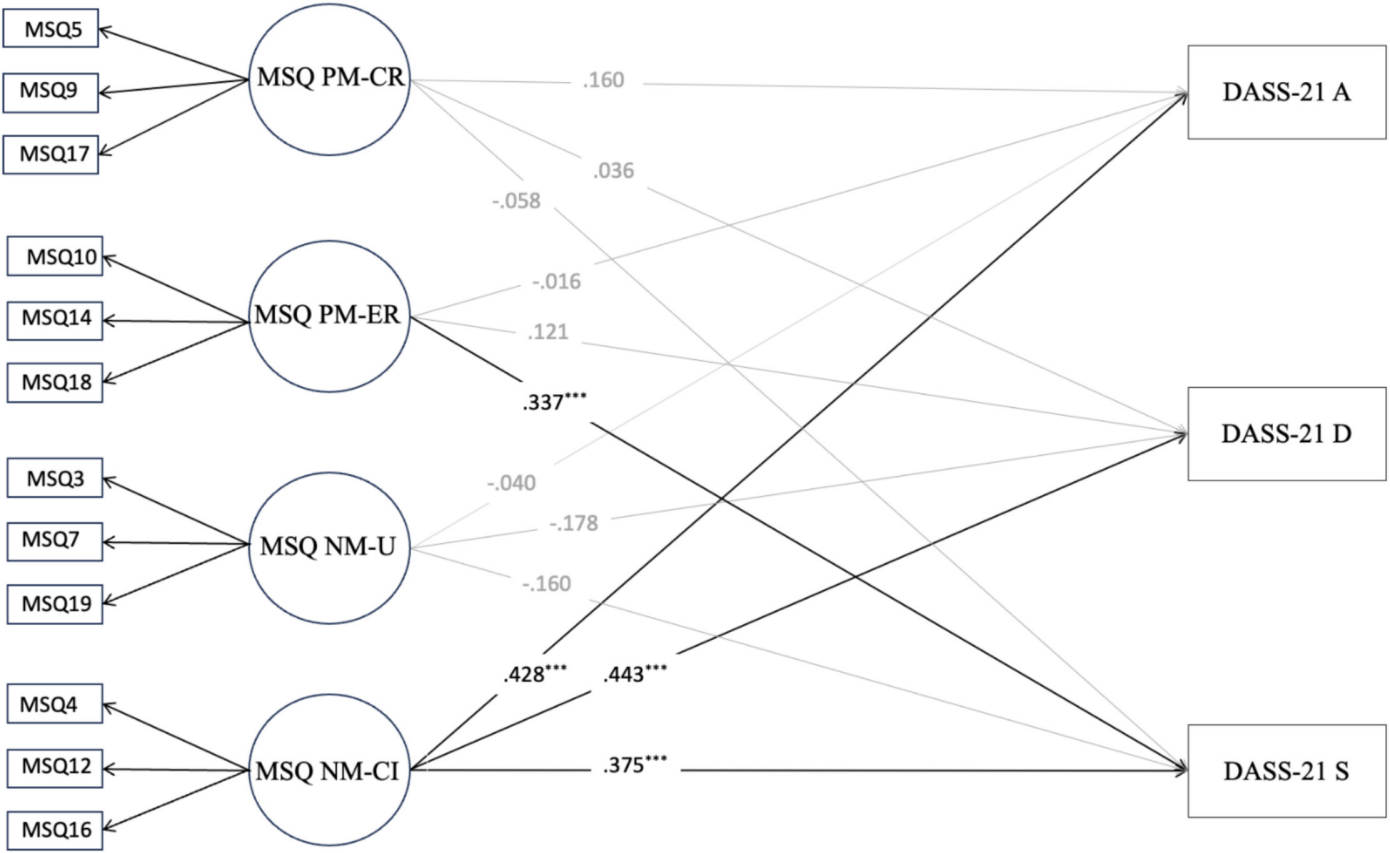
Structural equation model of the association between the MSQ four factors model and DASS‐21 anxiety, DASS‐21 depression and DASS‐21 stress. *Note: N* = 532. All structural coefficients are standardized. Model fit: TLI = 0.966, CFI = 0.976, RMSEA = 0.045 [0.035–0.055]. DASS‐21 A = Depression Anxiety Stress Scales—Anxiety subscale, DASS‐21 D = Depression Anxiety Stress Scales—Depression subscale, DASS‐21 S = Depression Anxiety Stress Scales—Stress subscale, MSQ NM‐CI = Metacognitions about Smoking Questionnaire—Negative metacognitions about cognitive interference, MSQ NM‐U = Metacognitions about Smoking Questionnaire—Negative metacognitions about uncontrollability, MSQ PM‐ER = Metacognitions about Smoking Questionnaire—Positive metacognitions about emotional regulation, MSQ PM‐CR = Metacognitions about Smoking Questionnaire—Positive metacognitions about cognitive regulation. All outcomes were controlled for gender.

Based on the CFA results, we tested the concurrent validity of the MSQ. Overall, the expected associations were found at the univariate level (Table 3). The subsequent SEMs showed good fit indices. Results revealed positive and significant associations between PM‐CR, NM‐U and nicotine dependence (FTND), and a significant and negative relationship between PM‐ER and nicotine dependence (FTND) (Figure 2). A significant positive association was also found between NM‐U and the severity of cigarette dependence (SDS) (see Figure 3). Results also revealed a positive and significant relationship between PM‐ER and stress (DASS‐21) (Figure 4). Similarly, a significant association was found between NM‐CI and anxiety, depression and stress (DASS‐21) (Figure 4).

**TABLE 3:** Pearson's intercorrelations of study variables.

	Mean (SD)	Range	MSQ PM‐CR	MSQ PM‐ER	MSQ NM‐U	MSQ NM‐CI	FTND	SDS	DASS‐21 A	DASS‐21 D	DASS‐S
**MSQ PM‐CR**	2.05 (0.83)	1–4	1								
**MSQ PM‐ER**	2.54 (0.81)	1–4	0.64***	1							
**MSQ NM‐U**	2.19 (0.84)	1–4	0.27***	0.36***	1						
**MSQ NM‐CI**	1.48 (0.74)	1–4	0.21***	0.25***	0.63***	1					
**FTND**	3.4 (2.22)	0–10	0.22***	0.15***	0.38***	0.31***	1				
**SDS**	1.58 (0.62)	0.2–3	0.12**	0.13**	0.36***	0.28***	0.58***	1			
**DASS‐21 A**	0.68 (0.47)	0–3	0.23***	0.20***	0.29***	0.39***	0.32***	0.35***	1		
**DASS‐21 D**	0.83 (0.65)	0–3	0.17***	0.18***	0.33***	0.33***	0.24***	0.22***	0.59***	1	
**DASS‐21 S**	1.08 (0.53)	0–3	0.23***	0.30***	0.31***	0.31***	0.14***	0.16***	0.61***	0.69***	1

*Note:* *N* = 532.

Abbreviations: DASS‐21 A = Depression Anxiety Stress Scales—Anxiety subscale, DASS‐21 D = Depression Anxiety Stress Scales—Depression subscale, DASS‐21 S = Depression Anxiety Stress Scales—Stress subscale, FTND = Fagerstrom Test for Nicotine Dependence, MSQ NM‐CI = Metacognitions about Smoking Questionnaire—Negative metacognitions about cognitive interference, MSQ NM‐U = Metacognitions about Smoking Questionnaire—Negative metacognitions about uncontrollability, MSQ PM‐CR = Metacognitions about Smoking Questionnaire—Positive metacognitions about cognitive regulation, MSQ PM‐ER = Metacognitions about Smoking Questionnaire—Positive metacognitions about emotional regulation, SDS = Severity Dependence Scale.

** *p* < 0.01.

*** *p* < 0.001.

**FIGURE 5:**
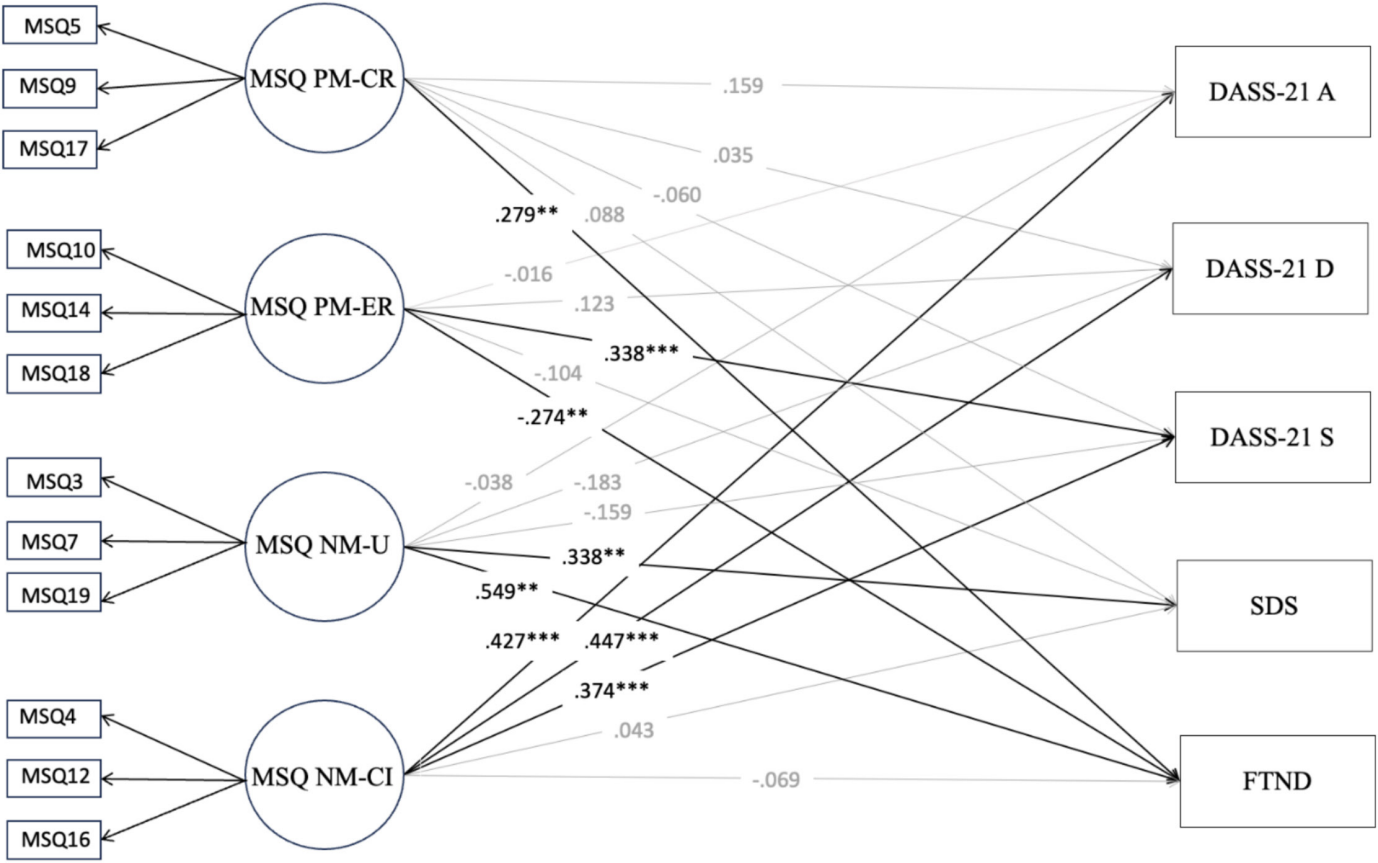
Structural equation model of the association between the MSQ four factors model and DASS‐21 anxiety, DASS‐21 depression, DASS‐21 stress, SDS severity of cigarette dependence and FTND nicotine dependence. *Note: N* = 532. All structural coefficients are standardized. Model fit: TLI = 0.959, CFI = 0.973, RMSEA = 0.046 [0.037–0.055]. DASS‐21 A = Depression Anxiety Stress Scales—Anxiety subscale, DASS‐21 D = Depression Anxiety Stress Scales—Depression subscale, DASS‐21 S = Depression Anxiety Stress Scales—Stress subscale, FTND = Fagerstrom Test for Nicotine Dependence, MSQ NM‐CI = Metacognitions about Smoking Questionnaire—Negative metacognitions about cognitive interference, SDS = Severity Dependence Scale, MSQ NM‐U = Metacognitions about Smoking Questionnaire—Negative metacognitions about uncontrollability, MSQ PM‐CR = Metacognitions about Smoking Questionnaire—Positive metacognitions about cognitive regulation, MSQ PM‐ER = Metacognitions about Smoking Questionnaire—Positive metacognitions about emotional regulation. All outcomes were controlled for gender.

When we examined the association between the MSQ dimensions and all external measures within the same model, SEM showed a similar pattern of results with an overall medium effect size (Figure 5). Specifically, PM‐CR was positively associated with nicotine dependence (FTND), while PM‐ER was negatively associated with nicotine dependence (FTND). Additionally, NM‐U was positively associated with both nicotine dependence (FTND) and severity of cigarette dependence (SDS). Results also revealed that a significant and positive association was found between PM‐ER and stress (DASS‐21). Likewise, positive and significant associations were found between NM‐CI and anxiety, depression and stress (DASS‐21).

Finally, internal consistency as assessed via Cronbach's alpha and McDonald's omega was satisfactory for all subscales (Table 4).

**TABLE 4:** Descriptive analyses and internal consistency of the MSQ.

		Mean	SD	Cronbach's alpha	McDonald's omega
1	PM‐CR	2.05	0.83	0.89	0.86
2	PM‐ER	2.54	0.81	0.84	0.80
3	NM‐U	2.19	0.84	0.77	0.76
4	NM‐CI	1.48	0.74	0.91	0.84

*Note:* *N* = 532. Cronbach's alpha and McDonald's omega were calculated based on the CFA model.

## 4 Discussion

Building on the metacognitive model of self‐regulation (Wells and Matthews 1996), and on preliminary research on metacognitions in smoking (Nikčević and Spada 2008, 2010; Spada et al. 2007), the present study aimed to evaluate the factor structure and psychometric properties of the Italian version of the MSQ (Nikčević et al. 2015) in a sample of Italian smokers. Contrary to previous studies run in a sample of adult smokers in the United Kingdom (Nikčević et al. 2015), Turkey (Alma et al. 2018) and Iran (Najafi et al. 2018), the initial four‐factor model including all 20 MSQ items showed an unsatisfactory fit. An inspection of the modification indices revealed that some items exhibited a multiproblematic aspect. This result could be attributable to cultural differences as well as to some translation issues. Consequently, several items included in the Italian version of the MSQ were removed. More in details items were removed based on both statistical and clinical‐theoretical considerations. Items that from a statistical point of view exhibited multiple problematic aspects (e.g., low loadings, cross‐loadings or redundancy), and that from a clinical perspective were deemed to have poor clarity, low salience or limited relevance, were removed. Based on this approach, and following a step‐by‐step procedure, seven items that exhibited cross‐loadings on more than one factor and/or multicollinearity, and were also deemed to have limited clinical relevance, were removed. Although the CFA conducted on the remaining 13 items demonstrated a good model fit, one additional item was subsequently removed based on clinical judgement, to obtain a more parsimonious version of the instrument. The Italian version of the MSQ resulted in the selection of 12 items divided in four factors i.e., PM‐CR (e.g., ‘Smoking helps me to order my thoughts’), PM‐ER (e.g., ‘Smoking distracts me from feeling pressure’), NM‐U (e.g., ‘I cannot control my urge to smoke’) and NM‐CI (e.g., ‘Thinking so much about smoking interferes with me seeing things clearly’) (Appendices A and B). The Italian version of the MSQ was found to possess a good fit, good convergent validity and a satisfactory discriminant validity of the constructs included in the model. Nevertheless, it should be noted that the relatively strong relationship between NM‐U and NM‐CI may indicate some conceptual overlap among these negative metacognitions. A previous study (Sumbe et al. 2024), conducted among US adolescent and young adult e‐cigarette users, proposed a broader dimension of ‘negative metacognition about smoking’ that integrates features of both NM‐U and NM‐CI. However, further research is needed to clarify the relationship between these two different types of negative metacognitions about smoking in adult cigarette smokers.

As been found elsewhere (Alma et al. 2018; Nikčević et al. 2015; Najafi et al. 2018), correlation analyses showed that all four MSQ factors were positively correlated with measures of nicotine dependence, severity of cigarette dependence and emotional distress, suggesting that the Italian version of the MSQ had good concurrent validity. Moreover, at multivariate levels, SEMs suggest that different metacognitions about smoking may have a different impact on clinical outcomes related to smoking.

As concern the association between metacognitions about smoking and nicotine/cigarette dependence, SEMs showed that higher endorsement on PM‐CR and on NM‐U are associated with higher levels of nicotine/cigarette dependence (i.e., SDS scores and FTND scores). These findings are in line with previous studies (Alma et al. 2018; Khosravani et al. 2024; Nikčević et al. 2015; Najafi et al. 2018). Consistent with the S‐REF model (Wells and Matthews 1996) applied to the addictive behaviours (Spada et al. 2013), it could be expected that a higher endorsement of PM‐CR may play a role in motivating individuals to engage in smoking as a means of cognitive regulation, while NM‐U may play a role in the perpetuation of smoking by becoming activated during and following a smoking episode (Nikčević and Spada 2010; Spada et al. 2013).

With regards to the association between metacognitions about smoking and emotional distress, SEMs showed that a higher endorsement of PM‐ER and NM‐CI is associated with more emotional distress. These results are aligned with the literature (Alma et al. 2018), suggesting that PM‐ER and NM‐CI may play a role in triggering and propagating distress and negative affect, which, in turn, are likely to bring about an increase in smoking (Cosci et al. 2019; Spada et al. 2013; Nikčević and Spada 2010).

Moreover, based on SEMs, a partial unexpected result of this study was that PM‐ER and NM‐CI were not associated with an increase in the levels of nicotine/cigarette dependence. Likewise, PM‐CR and NM‐U were not associated with an increase in emotional distress. These findings are aligned with the suggestions that some metacognitions could be more critical than others (Mansueto et al. 2022; Sun et al. 2017) in relation to poor clinical outcomes among smokers (Khosravani et al. 2024). For example, as observed by Khosravani et al. (2024), in male‐dependent smokers, among different metacognitions about smoking, only NM‐U was found to be associated with higher smoking dependence. However, further studies are required to clarify this issue in depth.

Finally, consistent with previous validation studies of the MSQ (Alma et al. 2018; Najafi et al. 2018; Nikčević et al. 2015), the Italian version of the MSQ was found to possess a satisfactory internal consistency for all subscales.

Overall, the final Italian version of the MSQ maintains the conceptual and clinical consistency with the original instrument (Nikčević et al. 2015), preserving the theoretical structure and clinical meaning of the original MSQ dimensions (Nikčević et al. 2015).

### 4.1 Clinical Implications

Our study findings bring us to consider their potential clinical implications. The Italian version of the MSQ could be a useful tool to enable clinicians and practitioners to identify and assess specific metacognitions about smoking among Italian smokers. Assessing metacognitions about smoking during treatment for smoking cessation would be an important therapeutic goal (Nikčević and Spada 2010). Smokers could be socialized about the association between PM‐CR and NM‐U on the one hand and the persistence of nicotine/cigarette dependence (Spada et al. 2013; Nikčević and Spada 2010) on the other. Furthermore, smokers could also be socialized that PM‐ER and NM‐CI are associated with higher emotional distress, which in turn may lead to the perseveration of smoking (Cosci et al. 2019; Spada et al. 2013; Nikčević and Spada 2010). Modifying positive and negative metacognitions about smoking could therefore be considered important therapeutic targets during smoking cessation. Metacognitive therapy (Wells 2000) would be a suitable approach to employ to reduce nicotine/cigarette dependence and emotional distress among smokers (Spada et al. 2013; Nikčević and Spada 2010).

### 4.2 Limitations

The present results are preliminary in nature. The most important limitation is the absence of a longitudinal study that precludes causal inferences. Moreover, the presence of concurrent psychological disorder and exposure to previous treatments aimed at modifying cognition was not assessed. An additional limitation concerns the model refinement process. The present study adopted a confirmatory approach while remaining open to exploratory adjustments, which represents an inherent methodological limitation. Although the analyses were conducted within a theory‐driven framework (Nikčević et al. 2015; Wells and Matthews 1996; Wells 2000), exploratory components were incorporated to improve model fit. The final 12‐item solution was informed by both theoretical assumptions and empirical evidence, including low factor loadings, cross‐loadings and item redundancy. The resulting structure demonstrated good global and local fit indices and reproduced conceptually coherent latent dimensions, consistent with previous works (Alma et al. 2018; Najafi et al. 2018; Nikčević et al. 2015). Nevertheless, given the flexible and semi‐exploratory nature of the analytic strategy, the proposed factor structure should be regarded as provisional and requiring further empirical validation. Future studies should aim to formally cross‐validate the model and examine its generalizability across independent and more heterogeneous samples, as well as across different cultures, to enhance the robustness of its psychometric properties. Moreover, future studies are required to further establish the psychometric properties of the Italian version of the MSQ and in particular test–retest reliability and predictive validity. Divergent validity could also be established by employing the MSQ during the smoking cessation treatment to discriminate between those who successfully stop smoking and those who do not. Furthermore, given that the current sample is inadequate for a stable and reliable estimation of measurement invariance, further studies are required to explore it in depth. Moreover, it should be considered that, on average, the participants included in the present study have moderate levels of nicotine/cigarette dependence. This may account for the lack of some significant associations identified. Future research should aim to replicate these findings among participants with higher levels of nicotine/cigarette dependence. Additionally, an examination of the psychometric properties of the Italian version of the MSQ among e‐cigarette users could also be carried out. Finally, further studies are required to explore the clinimetric properties of the Italian version of the MSQ (Carrozzino et al. 2021; Cosci 2021; Feinstein 1983; Mansueto et al. 2021).

## 5 Conclusion

The Italian version of the MSQ may represent a suitable clinical tool that may help clinicians and practitioners assess specific metacognitions about smoking. Given that higher endorsement of metacognitions about smoking may be associated with more severe nicotine/cigarette dependence and emotional distress, assessing metacognitions about smoking may allow clinicians to gain a clearer understanding of treatment trajectories among smokers (Khosravani et al. 2024; Nikčević and Spada 2010). Metacognitions about smoking may be a suitable therapeutic target to reduce the levels of nicotine/cigarette dependence and emotional distress among smokers (Nikčević and Spada 2010; Spada et al. 2013).

## Author Contributions

**Sara Palmieri:** data curation, writing – original draft. **Ana Nikčević:** writing – review and editing. **Tatiana Marci:** formal analyses. **Claudia Marino:** data curation. **Gabriele Caselli:** supervision. **Marcantonio M. Spada:** supervision. **Giovanni Mansueto:** conceptualization, writing – original draft, writing – review, methodology and supervision.

## Funding

The authors received no specific funding for this work.

## Ethics Statement

The study was conducted in accordance with the Declaration of Helsinki, and the protocol was approved by the Ethics Committee of the Sigmund Freud University. Informed consent was obtained from all individual subjects participating in the study.

## Conflicts of Interest

Two of the authors (i.e., Ana Nikčević and Marcantonio M. Spada) were involved in developing the original version of the MSQ for English‐speaking populations. The authors have no known competing financial interests or personal relationships that could have influenced this work.

## Supporting information


**Table S1:** Heterotrait–monotrait ratio of correlations.

## Data Availability

The data that support the findings of this study are available from the corresponding author upon reasonable request.

## Appendix A Adapted Italian Version of the Metacognitions About Smoking Questionnaire Short Form (MSQ‐SF)

Il presente questionario riguarda le credenze che le persone hanno riguardo al fumo. Di seguito sono elencate una serie di credenze che le persone hanno espresso. Per prima cosa, prova a pensare a quando fumi. Poi leggi ciascuna frase e indica quanto in generale sei d'accordo con essa cerchiando il numero appropriato. Per favore, rispondi a tutti gli items. Non ci sono risposte giuste o sbagliate.

**Table:**

		Non d'accordo	Leggermente d'accordo	Moderatamente d'accordo	Molto d'accordo	Fattore
1.	Fumare significa avere poca forza di volontà	1	2	3	4	NM‐U
2.	Pensare così tanto al fumo interferisce con la mia lucidità mentale	1	2	3	4	NM‐CI
3.	Fumare mi aiuta a focalizzarmi sulla mia mente	1	2	3	4	PM‐CR
4.	E' difficile controllare il mio desiderio di sigarette	1	2	3	4	NM‐U
5.	Fumare mi aiuta a mettere ordine nei miei pensieri	1	2	3	4	PM‐CR
6.	Fumare mi conforta quando sono turbato	1	2	3	4	PM‐ER
7.	I pensieri legati al fumare stanno diventando un'ossessione	1	2	3	4	NM‐CI
8.	Fumare mi aiuta a distendermi	1	2	3	4	PM‐ER
9.	La mia preoccupazione per le sigarette prende il sopravvento sulla mia vita	1	2	3	4	NM‐CI
10.	Fumare mi aiuta a concentrarmi	1	2	3	4	PM‐CR
11.	Fumare mi distrae dal sentirmi sotto pressione	1	2	3	4	PM‐ER
12.	Non riesco a controllare il bisogno di fumare	1	2	3	4	NM‐U

Abbreviations: NM‐CI = metacognizioni negative rispetto all'interferenza cognitiva, NM‐U = metacognizioni negative rispetto all'incontrollabilità, PM‐CR = metacognizioni positive rispetto alla regolazione cognitiva, PM‐ER = metacognizioni positive rispetto alla regolazione emotiva.

## Appendix B Metacognitions About Smoking Questionnaire Short Form (MSQ‐SF)

This questionnaire is concerned with beliefs people hold about smoking. Listed below are a number of beliefs that people have expressed. First, try to think about when you smoke. Then, read each item and determine how much you generally agree with it by circling the appropriate number. Please respond to all the items. There are no right or wrong answers.

**Table:**

		Do not agree	Agree slightly	Agree moderately	Agree very much	Factor
1.	Smoking means I have low will power	1	2	3	4	NM‐U
2.	Thinking so much about smoking interferes with me seeing things clearly	1	2	3	4	NM‐CI
3.	Smoking helps me to focus my mind	1	2	3	4	PM‐CR
4.	It is hard to control my desire for cigarettes	1	2	3	4	NM‐U
5.	Smoking helps me to order my thoughts	1	2	3	4	PM‐CR
6.	When I get upset smoking comforts me	1	2	3	4	PM‐ER
7.	My thoughts about smoking are becoming an obsession	1	2	3	4	NM‐CI
8.	Smoking helps me to unwind	1	2	3	4	PM‐ER
9.	My preoccupation with cigarettes takes over my life	1	2	3	4	NM‐CI
10.	Smoking helps me concentrate	1	2	3	4	PM‐CR
11.	Smoking distracts me from feeling pressured	1	2	3	4	PM‐ER
12.	I cannot control my urge to smoke	1	2	3	4	NM‐U

Abbreviations: NM‐CI = Negative metacognitions about cognitive interference, NM‐U = Negative metacognitions about uncontrollability, PM‐CR = Positive metacognitions about cognitive regulation, PM‐ER = Positive metacognitions about emotional regulation.
