# Supplementary material for: Metacognitions About Smoking: Psychometric Properties of the Italian Version of the Metacognitions About Smoking Questionnaire
Source: Clin Psychol Psychother. 2025 Dec 22;32(6):e70208. doi: 10.1002/cpp.70208 (PMC12720064; doi:10.1002/cpp.70208)
Supplement: Supplementary file 1 — Table S1: Heterotrait–monotrait ratio of correlations. [file CPP-32-e70208-s001.docx]

**Table S1. Heterotrait-monotrait ratio of correlations**

|  | **PM-CR** | **PM-ER** | **NM-U** | **NM-CI** |
| --- | --- | --- | --- | --- |
| **PM-CR** | **-** | **.802** | **.347** | **.303** |
| **PM-ER** |  | **-** | **.477** | **.377** |
| **NM-U** |  |  | **-** | **.894** |
| **NM-CI** |  |  |  | **-** |

**Note.** HTMT values were used to assess discriminant validity among factors. Values below 0.85 (or less stringent thresholds such as 0.90, see Henseler et al., 2015) indicate adequate discriminant validity. PM-CR = Positive metacognitions about cognitive regulation, PM-ER = Positive metacognitions about emotional regulation, NM-U = Negative metacognitions about uncontrollability, NM-CI = Negative metacognitions about cognitive interference
